# A prospective cross-over study to evaluate the effect of two different occlusal concepts on the masseter muscle activity in implant-retained mandibular overdentures

**DOI:** 10.1186/s40729-015-0034-y

**Published:** 2015-12-22

**Authors:** Ahmed M. Abdelhamid, Kenda I. Hanno, Mohamed H. Imam

**Affiliations:** 1 Prosthodontics Department, Faculty of Dentistry, Alexandria University, Alexandria, Egypt; 2 Prosthodontics, Faculty of Dentistry, Alexandria University, Alexandria, Egypt; 3 Physical Medicine, Rheumatology and Rehabilitation, Faculty of Medicine, Alexandria University, Alexandria, Egypt

**Keywords:** Balanced occlusion, Canine guidance, Electromyography, Implant-retained overdentures, Muscle activity

## Abstract

**Background:**

The purpose of this prospective cross-over study is to evaluate the effect of bilateral balanced occlusion and canine guidance occlusion on the masseter muscle activity using implant-retained mandibular overdentures.

**Methods:**

After evaluation of 12 completely edentulous patients using cone beam computed tomography (CBCT), mucoperiosteal flaps were reflected exposing the mandibular interforaminal region. Two implants were placed in the interforaminal region for each of the 12 patients. After a healing period of 3 months, acrylic maxillary complete dentures and mandibular overdentures were fabricated with bilateral balanced occlusion for 6 patients and canine guidance occlusion for the other 6 patients. Electromyographic evaluation of the masseter muscles, during clenching on a silicon index and chewing peanuts and cake, was conducted on the patients after using their dentures for 4 weeks. Each occlusion concept was then converted into the other concept using the same dentures, and the procedure of evaluation was repeated after 4 weeks. The recordings were analyzed statistically using Wilcoxon signed ranks test. *p* < 0.05 was considered statistically significant.

**Results:**

The highest electromyographic activity of the masseter muscles was recorded during clenching on a preformed silicon index followed by chewing peanut then cake for both occlusal concepts. The recordings of the masseter muscle associated with canine guidance occlusion were higher than bilateral balanced occlusion but with no statistically significant difference except between the right masseter muscles during clenching (*p* = 0.042*).

**Conclusions:**

Both bilateral balanced occlusion and canine guidance occlusion can be used successfully in implant-retained mandibular overdentures without affecting masseter muscle activity.

## Background

Treatment of edentulous patients using a conventional complete removable denture is a common clinical undertaking. However, those patients may experience problems which include pain during mastication, reduced masticatory ability, as well as insufficient stability and retention of the mandibular denture [1].

The introduction of dental implants and, subsequently, the implant-supported mandibular overdenture has improved the quality of life for edentulous patients [2]. The biomechanical aspects of occlusal design, configuration, and anatomy significantly influence the ultimate success of implant. The development of occlusal concepts that is in harmony with the rest of the stomatognathic system is a major contributing factor in the long-term success rate of the implant-supported prosthesis [3].

Many authors [4] emphasized the role of occlusion as a key factor in implant success. James [5] stated that “nearly all problems of the implants that develop between the first and sixth week post-insertion are directly related to the occlusion.” Lindquist et al., [6] and Miyata et al., [7] studied the longitudinal effects of occlusal forces on osseointegrated implants and found that overloading was the main cause for bone loss around fixtures and/or loss of osseointegration of successfully integrated implants. The prosthesis must therefore be fabricated as accurately as possible in order to achieve long-term success, and occlusion should be a key factor of the overall success rate.

Currently, the occlusal philosophies that are proposed for implant overdentures are based on those for conventional complete dentures [8]. These philosophies include bilateral balanced occlusion and lingualized occlusion.

Balanced occlusion concept has been recommended in removable implant overdentures especially for patients with complete maxillary denture and mandibular implant-retained overdentures to distribute the loads and to provide better stability [9]. The chewing forces are applied to the working and balancing sides simultaneously and over all of the surfaces of the edentulous ridge, resulting in the decrease in pressure on the edentulous jaw and preventing residual ridge reduction [10].

For more than a hundred years, textbooks and undergraduate teaching have stated that canine guided dentures should be avoided, since they would result in denture instability and impaired muscle function; however, recent research has shown that conventional complete dentures can function successfully without balanced occlusion [11].

Canine guidance disengages the posterior teeth during excursive mandibular movements by vertically and horizontally overlapping the canines [12]. Grubwieser et al., [13] stated that canine guidance occlusion (CGO) can be used successfully in complete dentures as it improves mandibular denture retention, esthetic appearance, and masticatory ability. They also observed that CGO reduced muscle activity during lateral movement and protrusion when compared with BBO.

Canine guidance is easier and faster to be provided. It seems to be rational to recommend this occlusal concept for the excursive movements of the mandible in complete dentures wearers until future research can solve this question [14].

The various methods for assessment of masticatory function include food particle size estimation [15], sieving method [16], colorimetric determination [17], optical scanning method [18], biting force [15], and weight loss of viscoelastic food [19].

Occlusion may alter muscle activity and related jaw movements in mastication. Muscle activity, which is a reflection of masticatory function of the patient, can be objectively evaluated using surface electromyography (EMG) [20]. EMG is a valuable parameter to assess muscular dysfunction. Surface EMG measures muscle activity noninvasively using surface electrodes placed on the skin overlying the muscle to determine the timing of the muscle contraction and to investigate the behavior of the muscle during functions of the stomatognathic system [21].

Aziz et al., (2008) compared the effect of two different occlusal schemes on the muscle activity in implant-supported mandibular overdentures using electromyography. The results revealed slight increase in the muscle activity in patients rehabilitated with dentures constructed following the lingualized concept of occlusion compared to dentures constructed following the monoplane concept of occlusion [22].

The choice of an occlusal design or configuration for implant-retained prosthesis is broad and often controversial. To date, there is no evidence to recommend a specific occlusal design for implant-retained overdentures. No sufficient scientific evidence was found on applying canine guidance in implant-retained overdentures. Therefore, this study was conducted to investigate the effects of balanced occlusion and canine guidance on masseter muscle activity in implant-retained overdentures.

## Methods

The study design is a prospective cross-over study.

Twelve completely edentulous patients aged 45–65 years, having acceptable maxillomandibular relationship, sufficient inter-ridge space, and with no previous denture experience, were selected for this study. Complete maxillary and mandibular dentures were fabricated for each patient.

Since the type of opposing occlusion is a critical factor that influences the magnitude of forces transmitted to the implant bone interface, the opposing occlusion was selected to be mucosa-supported complete denture. This was done to standardize and control the amount of occlusal forces applied to the abutments. Complete dentures were proved to exert less amount of force compared to the natural teeth [23].

The selection of the interforaminal area of the mandible, where two implants were placed, was based on the recommendation by Lekholm and Zarb [24] and Hong et al., [25] as the bone in this area is of good quality. Implants are demonstrated to have fewer micro-movements, increased initial stability, and reduced stress concentration in high-quality bone [26]. Furthermore, it has been established that the survival of the root form titanium implants is very high in the anterior mandible and that the incidence of surgical complications is very low [27, 28].

A clear acrylic radiographic/surgical mandibular template including gutta-percha radiopaque indicators allowed implant alignment along planned prosthetic axes during implant surgery and ensured good visual access [29].

The previously fabricated denture was duplicated in clear acrylic resin and used as a radiographic surgical guide using gutta-percha as radiopaque markers. Each patient was evaluated radiographically using cone beam computed tomography (CBCT) before surgical implant placement. A mucoperiosteal flap was reflected exposing the mandibular interforaminal region for optimal implant placement. Two implants (Dentium Superline, Dentium Co. Ltd., Korea) were screwed in position. The length of the implants was 10 mm and the diameter is 3.6 mm.

After a healing period of 3 months, acrylic maxillary and mandibular overdentures were fabricated with bilateral balanced occlusion for all patients. Positioner attachments were used and incorporated into the dentures using direct pickup method. Six patients used their dentures with bilateral balanced occlusion for 4 weeks then were evaluated by EMG. Following evaluation, it was converted into canine guidance occlusion using the same denture. This was achieved clinically by remounting using the semi-adjustable articulator.

Light-cured composite resin was then added in the mandibular canines to provide an interarch disocclusion space of 2 mm during eccentric movements.

**Fig. 1:**
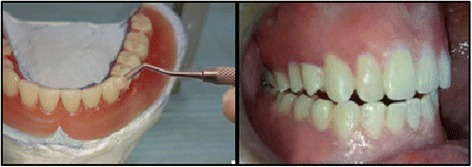
Application of light-cure composite resin on the mandibular canines and a view of canine guidance occlusion intraorally

For the other six patients, canine guidance occlusion was applied first in the try-in stage through application of light-cure composite resin on the mandibular canines (Fig. 1).

**Fig. 2:**
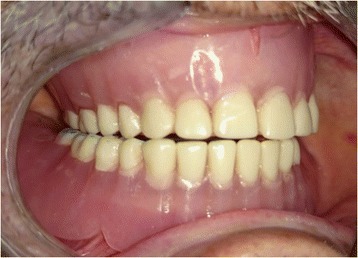
Bilateral balanced occlusion intraorally

**Fig. 3:**
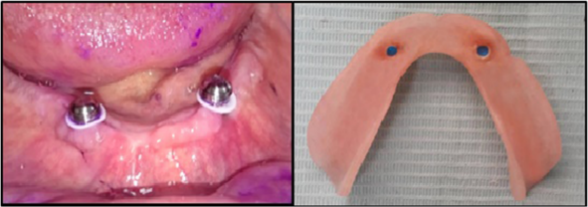
The white blockout spacers in place with the locator caps on top and the positioner socket with blue nylon cap

The patients used their dentures for 4 weeks then were evaluated using EMG. After evaluation, canine guidance occlusion was converted into bilateral balanced occlusion by removal of the composite resin from the mandibular canines (Fig. 2). The positioner attachments (positioner abutment and socket set, Dentium Co.Ltd, Korea) were placed on the implants and attached to the dentures by direct pickup method (Fig. 3).

### Recording the electromyographic activity

EMG has also been used to assess the masticatory function of mandibular implant-retained overdentures. The electrical output of a muscle, measured by electromyography, is proportional to the energy consumed to produce contractions. The masseter and anterior temporalis muscles on both sides were evaluated because they are the largest and strongest of the masticatory muscles, the most superficial and are accessible to surface EMG examination. The surface EMG recordings provided a safe, easy, and noninvasive method that allowed objective quantification of the energy of the muscle [30].

Standard amounts and sizes of cake and peanuts were used to reduce patient variability. These test foods were an example of soft and hard food, respectively, and they gave an idea about the effect of different types of food on muscle activity during function. Preformed silicon index was used to measure the muscle activity during clenching [31].

Evaluation of muscle activity was performed by measuring activity of the masseter muscles on both sides for both occlusal concepts at the end of 2 weeks using electromyography (Nicolet VikingQuest version 11, USA) with three types of test foods.

**Fig. 4:**
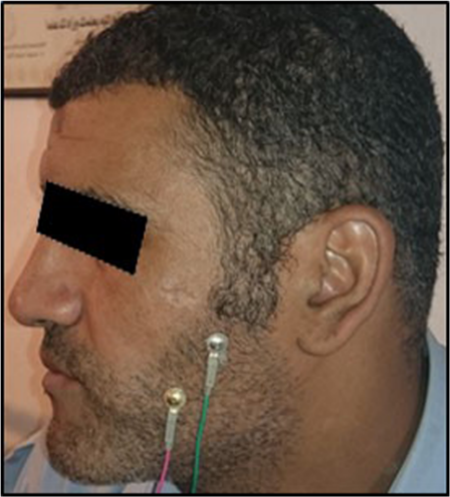
Bipolar electrodes positioned at the masseter muscle belly

During all recordings, the patients were seated with their head unsupported and were asked to maintain a naturally erect position. The masseteric myoelectric activity of both sides (right and left) were recorded by means of disposable bipolar electrodes positioned on the bellies of the muscles parallel to the fiber orientation (Fig. 4).

Electroconductive gel was applied on the electrodes before they contacted the skin. The recording electrodes were positioned approximately 20 mm apart. The patient was grounded by using a grounding electrode. The third electrode was fixed on the palm of the patient’s hand.

Each patient was instructed to clench with a preformed silicon index made by using vinyl polysiloxane material (putty) of standardized size positioned at the premolar region for 30 s to measure the muscle activity during clenching. Then the patients were instructed to chew on one peanut of similar size and diameter, and the EMG was recorded. Then the patients were instructed to chew on a piece of cake of standardized size (2 cm × 2 cm), and the EMG was recorded. The patients chewed the test samples on the right and left sides at 10-s intervals using their arbitrary chewing frequency until they are ready to swallow before the EMG was recorded.

**Fig. 5:**
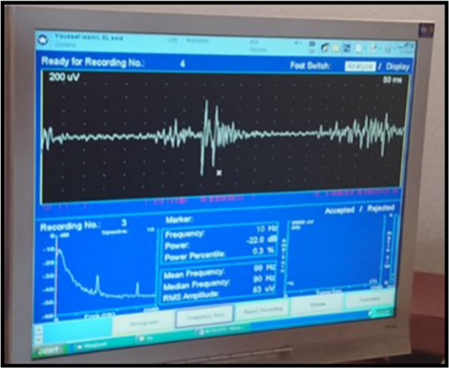
EMG signal on the monitor of the computer

At the end of the record and before removing the surface electrodes, the positions of the electrodes were marked to be used as a guide for accurate reproducibility. The previous tasks were separated by a recovery rest period of 2 min. The computerized data showed the root mean square (RMS) of the EMG signals (Fig. 5).

### Statistical analysis

Data were fed to the computer. Quantitative data were described using range (minimum and maximum), mean, standard deviation, and median. Wilcoxon signed ranks test was applied. Significance of the obtained results was judged at the 5 % level. A *p* value less than 0.05 was considered statistically significant.

### Ethical approval

This study protocol was approved by the research ethics committee of the Faculty of Dentistry, Alexandria University, Egypt.

### Ethics, consent, and permissions

All the patients signed an informed consent form before participation in this study.

### Consent to publish

All the patients who participated in the study provided consent to publish the data obtained from them during the study.

## Results

In the right masseter muscle, bilateral balanced occlusion has shown lower RMS values on clenching with the silicon index, chewing peanut, and chewing cake compared to the RMS values of canine guidance occlusion (Table 1).

**Table 1:**
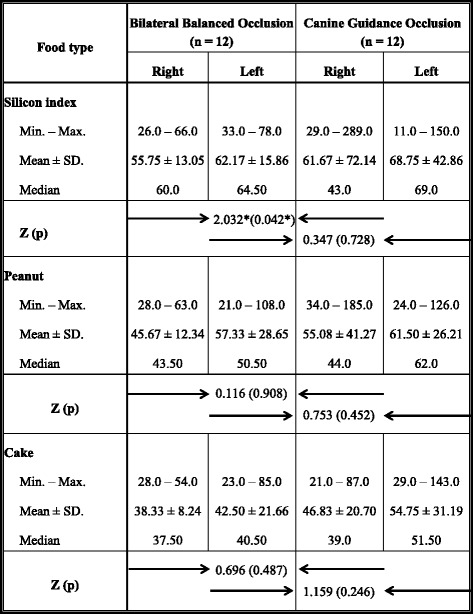
Comparison between bilateral balanced occlusion and canine guidance occlusion according to EMG signals (RMS values) of the masseter muscle

*Z* Mann-Whitney test

*Statistically significant at *p* ≤ 0.05

However, the table shows no statistically significant difference between the two occlusal concepts for the right masseter muscle. The difference was statistically significant only during clenching on a silicon index (*p* = 0.042*).

In the left masseter muscle, bilateral balanced occlusion has shown lower RMS values on clenching with the silicon index, chewing peanut, and chewing cake compared to the RMS values of canine guidance occlusion. The table shows no statistically significant difference between the two occlusal concepts for the left masseter muscle (Table 1).

**Table 2:** Comparison between the different test foods according to RMS values of the EMG signals of right and left masseter muscles in bilateral balanced occlusion

	Silicon index (*n* = 12)	Peanut (*n* = 12)	Cake (*n* = 12)	^KW^ *χ* ^2^	*p*
Right					
Min.–Max.	26.0–66.0	28.0–63.0	28.0–54.0		
Mean ± SD	55.75 ± 13.05	45.67 ± 12.34	38.33 ± 8.24	10.180^*^	0.006^*^
Median	60.0	43.50	37.50		
	*p*_1_ = 0.005^*^, *p*_2_ = 0.037^*^, *p*_3_ = 0.063				
Left					
Min.–Max.	33.0–78.0	21.0–108.0	23.0–85.0		
Mean ± SD	62.17 ± 15.86	57.33 ± 28.65	42.50 ± 21.66	5.013	0.082
Median	64.50	50.50	40.50		
	*p*_1_ = 0.037^*^, *p*_2_ = 0.354, *p*_3_ = 0.131				

^*KW*^ *χ* ^*2*^ chi square for Kruskal-Wallis test, *p*_*1*_ *p* value for Mann-Whitney test for comparing between Silicon index and Cake, *p*_*2*_ *p* value for Mann-Whitney test for comparing between silicon index and peanut, *p*_*3*_ *p* value for Mann-Whitney test for comparing between cake and peanut

*Statistically significant at *p* ≤ 0.05

In the right masseter muscle, clenching on silicon index showed the highest RMS values followed by chewing peanut, and the least RMS value was for chewing cake. The results showed a statistical significant difference between them (*p* = 0.006).

In the left masseter muscle, clenching on silicon index showed the highest RMS values followed by chewing peanut and the least RMS value was for chewing cake. The results showed no statistical significant difference between them (*p* = 0.082). There was a statistical significant difference between clenching on silicon index and chewing cake (*p*_1_ = 0.037^*^) (Table 2).

**Table 3:** Comparison between the different test foods according to RMS values of the EMG signals of right and left masseter muscles in canine guidance occlusion

	Silicon index (*n* = 12)	Peanut (*n* = 12)	Cake (*n* = 12)	^KW^ *χ* ^2^	*p*
Right					
Min.–Max.	29.0–289.0	34.0–185.0	21.0–87.0		
Mean ± SD	61.67 ± 72.14	55.08 ± 41.27	46.83 ± 20.70	0.501	0.778
Median	43.0	44.0	39.0		
	*p*_1_ = 1.000, *p*_2_ = 0.488, *p*_3_ = 0.602				
Left					
Min.–Max.	11.0–150.0	24.0–126.0	29.0–143.0		
Mean ± SD	68.75 ± 42.86	61.50 ± 26.21	54.75 ± 31.19	2.134	0.344
Median	69.0	62.0	51.50		
	*p*_1_ = 0.225, *p*_2_ = 0.728, *p*_3_ = 0.202				

^*KW*^ *χ* ^*2*^ chi square for Kruskal-Wallis test, *p*_*1*_ *p* value for Mann-Whitney test for comparing between silicon index and cake, *p*_*2*_ *p* value for Mann-Whitney test for comparing between silicon index and peanut, *p*_*3*_ *p* value for Mann-Whitney test for comparing between cake and peanut

*Statistically significant at *p* ≤ 0.05

In the right masseter muscle, clenching on silicon index showed the highest RMS values followed by chewing peanut and the least RMS value was for chewing cake. The results showed no statistical significant difference between them (*p* = 0.778).

In the left masseter muscle, clenching on silicon index showed the highest RMS values followed by chewing peanut, and the least RMS value was for chewing cake (54.75 ± 31.19). The results should no statistical significant difference between them (*p* = 0.344). (Table 3)

## Discussion

Many authors stated that bilateral balanced occlusion promotes better masticatory efficiency by bringing a larger amount of grinding surfaces into contact at each movement [32, 33]. The results of the current study did not show a significant difference for muscle function by EMG between the studied occlusal concepts. This finding coincides with El-Okel who compared masticatory efficiency through evaluation of electromyographic activity between bilateral balanced occlusion, canine guidance occlusion, lingualized, and monoplane occlusion schemes and found no significant difference [34]. The results also coincide with Grunert et al., [20] who found no difference in muscle activity between bilateral balanced occlusion and canine guidance occlusion in complete dentures. These results were also similar to Neto AF et al., who stated that bilateral balanced occlusion did not improve masticatory efficiency when compared with canine guidance [11]. Abd Elmonem evaluated masticatory efficiency in complete denture wearers with bilateral balanced occlusion and canine guidance using the colorimetric method and also found no statistical significant difference between them [35].

Despite the insignificant difference between EMG activity of the masseter muscles in bilateral balanced occlusion and canine guidance occlusion, the mean EMG activity was higher in canine guidance compared to bilateral balanced occlusion. This could be due to larger amount of grinding surfaces brought into contact at each movement in balanced occlusion resulting in less effort needed by the muscles during chewing. This could also be due to bilateral balanced occlusion facilitating adaptation to the new denture as stated by Rehmann et al. [36]. These results are in contrast with Grubweiser et al., [13] and Miralles et al., [37] who found that canine guidance occlusion reduced masseter muscle activity in complete denture wearers. They stated that neuromuscular function in edentulous subjects is similar to that found in dentate people and that canine guided dentures prevent parafunctional habits.

The results of this study showed that the EMG activity of the masseter muscles during clenching on a preformed silicon index was significantly higher than during chewing peanut and chewing cake. This finding is in line with Miralles et al., [37] who pointed out that the increased EMG activity may be due to increased vertical dimension leading to increased muscle activity during maximum voluntary clenching. This finding is also in line with Darwish et al., [38]. The results also show higher mean EMG activity when chewing peanut than when chewing cake in this study. This coincides with van der Bilt et al., [39] who stated that harder food consistency required higher muscle activity levels due to higher muscle force needed to comminute hard food. The results are also in agreement with Karkazis [40] who found that harder foods required higher chewing rates, higher electrical activity of the masseter muscle, and higher relative contraction periods, accompanied by shorter cycle durations.

### Limitations of the study

These include short follow-up periods, no subjective assessment of the dentures with different occlusion concepts using questionnaires and depending entirely on electromyography for the assessment of masticatory function.

## Conclusions

Within the limitations of this study of short follow-up periods of mandibular implant-retained overdenture and maxillary complete denture, the results lead to that both bilateral balanced occlusion and canine guidance occlusion can be used successfully in implant-retained overdentures without affecting masticatory function. However, the procedures involved in construction of dentures with bilateral balanced occlusion are more complex and time consuming. Greater muscle activity of both muscles is shown when chewing hard food due to higher muscle force needed to comminute hard food compared to soft food.

### Recommendations

The present study recommends the following:

- Further investigations for a longer follow-up period and for a larger sample to confirm widespread use of canine guidance occlusal concept and involving subjective assessment using questionnaires for the patients’ satisfaction
- Evaluation of the effect of different attachment types on masticatory function using the canine guidance concept in implant-retained overdentures
- Evaluation of canine guidance occlusal concept using a different method of evaluation of masticatory function other than electromyography

## Abbreviations

BBO: Bilateral Balanced Occlusion
CBCT: Cone Beam Computed Tomography
CGO: Canine Guidance Occlusion
EMG: Electromyography
ME: Masticatory Efficiency
